# Determination of Accuracy of Fetal Weight Using Ultrasound and Clinical Fetal Weight Estimations in Calabar South, South Nigeria

**DOI:** 10.1155/2014/970973

**Published:** 2014-11-10

**Authors:** Charles Njoku, Cajethan Emechebe, Patience Odusolu, Sylvestre Abeshi, Chinedu Chukwu, John Ekabua

**Affiliations:** ^1^Department of Obstetrics & Gynecology, University of Calabar Teaching Hospital (UCTH), PMB 1278, Calabar, Cross River State, Nigeria; ^2^Department of Radiology, University of Calabar Teaching Hospital (UCTH), Calabar, Cross River State, Nigeria

## Abstract

Information on fetal weight is of importance to obstetricians in the management of pregnancy and delivery. The objective of this study is to compare the accuracy of clinical and sonographic methods of predicting fetal weights at term. This prospective comparative study of 200 parturients was conducted at the University of Calabar Teaching Hospital, Calabar. The study participants were mothers with singleton term pregnancy admitted for delivery. The mean absolute percentage errors of both clinical and ultrasound methods were 11.16% ± 9.48 and 9.036% ± 7.61, respectively, and the difference was not statistically significant (*P* = 0.205). The accuracy within 10% of actual birth weights was 69.5% and 72% for both clinical estimation of fetal weight and ultrasound, respectively, and the difference was not statistically significant (*P* = 0.755). The accuracy of fetal weight estimation using Dare's formula is comparable to ultrasound estimates for predicting birth weight at term.

## 1. Introduction

Assessment of fetal weight is a vital and universal part of antenatal care, not only in the management of labor and delivery but often during the management of high risk pregnancies and growth monitoring [1]. Birth weight of an infant is the single most important determinant of newborn survival [1, 2]. Both low and excessive fetal weights at delivery are associated with an increased risk of newborn complications during labor and puerperium. The high perinatal morbidity and mortality associated with low birth weight are attributable to preterm delivery, intrauterine growth restriction, or both. For excessively large fetuses, the potential complications associated with vaginal delivery include shoulder dystocia, brachial plexus injury, bone injuries, and intrapartum asphyxia, while the maternal risks include birth canal and pelvic floor injuries, increased rate of operative vaginal and caesarean deliveries, and postpartum haemorrhage [3]. Limiting the potential complications associated with the birth of both small and excessively large fetuses requires that accurate estimation of fetal weight occurs before decision to deliver is made [4]. The two main methods for predicting birth weight in current obstetrics are clinical and ultrasonographic methods [5, 6]. Increasing attention is being paid to the accuracy of using various ultrasound measurements in estimating fetal weight. Multiple fetal parameters for prediction of fetal weight are employed. These are the biparietal diameter, head circumference, abdominal circumference, and femoral length. Ultrasound estimation of fetal weight, while being accurate to a degree, is associated with error ranging from ±6 to 11% depending on parameters measured and the equation used for estimation [7]. Although some investigators consider sonographic estimates to be superior to clinical estimates, others in comparing both techniques concurrently concluded that they confer similar level of accuracy [8, 9]. In developing countries, it is important to note that ultrasound fetal weight estimation requires expensive equipment and trained personnel and is time-consuming, while clinical methods can be carried out at no cost and are easy to perform especially for less experienced examiners [1, 10]. The aim of this study is to determine which method of fetal weight estimation (clinical or sonographic) is more accurate. This will help in appropriate decision making in the management of the pregnant woman.

## 2. Methodology

### 2.1. Material and Methods

This prospective cross-sectional comparative study was carried out at the Obstetrics and Gynecology Department of the University of Calabar Teaching Hospital (UCTH), Calabar. The study population was mothers with singleton term pregnancy in cephalic presentation, admitted for either normal vaginal delivery, elective caesarean section, or induction of labor. The participants had their gestational ages confirmed by an early ultrasound scan before 22 weeks of gestation depending on when they booked for antenatal care. Follow-up care was according to departmental protocols. Exclusion criteria included unbooked women, polyhydramnios, preterm labor, ruptured membranes, abnormal lie and presentation, multiple pregnancies, antepartum haemorrhage and eclampsia, obvious congenital anomaly, oligohydramnios, uterine fibroids, anteriorly inserted placenta, and poor visualization of fetal parts. A total of 200 mothers participated in the study over a 5-month period from May 16, 2013, to October 30, 2013. The selection was done using systematic random sampling. The first participant was randomly selected and every third participant who satisfied the inclusion criteria was recruited into the study. The interval between clinical and ultrasound estimation of fetal weight in-utero and delivery of the babies was within 72 hrs. Information on age, last menstrual period, gestational age, and parity was obtained from participants and case files before delivery. The maternal weight was determined using adult weighing scale with minimal clothing and recorded. Then in-utero clinical estimations of fetal weight were carried out in the labor ward using a flexible tape measure calibrated in centimeter. The fundal height was measured from the highest point on the uterine fundus to the midpoint of the upper border of the symphysis pubis. The abdominal circumference was measured at the level of the umbilicus. Dare formula (fundal height multiplied by the abdominal circumference in centimeters) was used to calculate the clinical fetal weights in grammes. After the clinical estimations, the patients had ultrasonographic estimations of fetal weight. The ultrasound machine used was real-time with abdominal sector 3.5 MHz transducer. The ultrasound machine formula for estimating fetal weight was that devised by Hadlock on the basis of biparietal diameter (BPD), head circumference (HC), abdominal circumference (AC), and femoral length (FL). Both clinical and ultrasonic estimates were documented in a chart. After delivery, the birth weights of the babies were determined within 30 minutes of delivery employing a standard analogue Waymaster (England) scale corrected for zero error. All data obtained during the study period were entered into a data form specifically designed for the study.

The mean birth weight and standard deviation from actual birth weight following clinical and ultrasonic methods of fetal weight estimations were calculated. The accuracy of clinical and sonographic estimation of fetal weight was determined with respect to the following: (1) mean of the error (EFW − ABW/ABW), (2) mean of absolute error (absolute value of EFW − ABW/ABW), (3) mean percentage error ([EFW − ABW] × 100/ABW), (4) mean of absolute percentage error (absolute value of [EFW − ABW] × 100/ABW), and (5) ratio (%) of estimates within 10% of actual birth weight (true when absolute percentage error is not more than 10%). Difference between both methods in the mean percentage error (i.e., the systematic error) in each method was assessed by the paired *t*-test. Because the absolute errors were not normally distributed, Wilcoxon signed-rank test (nonparametric method) was used to test the differences between clinical and ultrasonic estimates. The difference in proportion of estimates that were within 10% of the actual birth weight was assessed by chi-square test. *P* < 0.05 was considered significant. All of the data analysis was done using Microsoft SPSS version-18, a windows based statistical program.

### 2.2. Limitations of the Study

The potential limitation of this study includes the use of only one sonographic model to derive estimates of fetal weights which may not be very accurate for all ranges of fetal weights. However the use of Hadlock formula which measures multiple fetal parameters was used which may reduce such errors.

### 2.3. Ethical Considerations

Information on the study was given to the participants who voluntarily decided whether or not to enroll on the study, after the approval by hospital Research and Ethics Committee. Informed consent was obtained from all participants before the study.

## 3. Results

Out of the 200 participants, there were 67 (33.5%) nulliparous women and 133 (66.5) multiparous women. A total of 36 (18%) delivered by caesarean section, while 164 (82%) delivered vaginally. The mean time between the fetal weight estimations and delivery was 47.36 ± 13.451 hours. The mean actual birth weight was 3,242 ± 508 g. The low birth weight babies (birth weight < 2,500 g) accounted for 12 (6.0%) and normal weight babies (birth weight 2,500 to <4,000 g) were 164 (82%), while macrosomic babies (birth weight ≥ 4,000 g) were 24 (12.0%).

The demographic characteristics of the study population are shown in Table 1. The mean maternal age was 28.86 ± 6.355 years and the median was 27 years (range 16–44 years). The mean maternal ages for low birth weight, normal birth weight, and macrosomic babies were 24.92 ± 5.854, 28.78 ± 6.580, and 31.42 ± 5.073 years, respectively, and their mean difference was not statistically significant (*P* = 0.060). The mean actual birth weight increased significantly with increase in both parity and maternal weight at delivery.

The mean actual birth weight was 3,242 ± 508 g, while the mean estimated fetal weights by clinical and ultrasound methods were 3,541 ± 633 g and 3,141 ± 441 g, respectively (Table 2). Paired *t*-test on mean ultrasonically calculated weight taken before birth of fetus and actual birth weight revealed no significant difference (*t* = 2.259, *P* = 0.122). It was also found that actual birth weight was not significantly different from clinically estimated weight (*t* = 0.453, *P* = 0.695).

**Figure 1:**
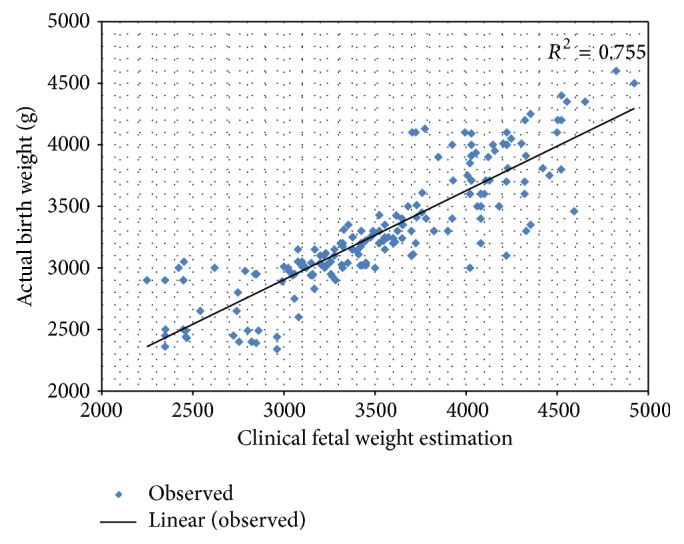
The scatter diagram of clinical fetal weight estimation and actual birth weight. Clinical fetal weight estimation showed positive correlation with the actual birth weight.

The scatter diagram showing the relationship between the clinical fetal weight estimation and actual birth weight is in Figure 1. Clinical method (Dares formula) of fetal weight estimation showed positive correlation with actual birth weight of the fetus after delivery. There is a positive linear relationship between clinical fetal weight estimation and actual birth weight.

**Figure 2:**
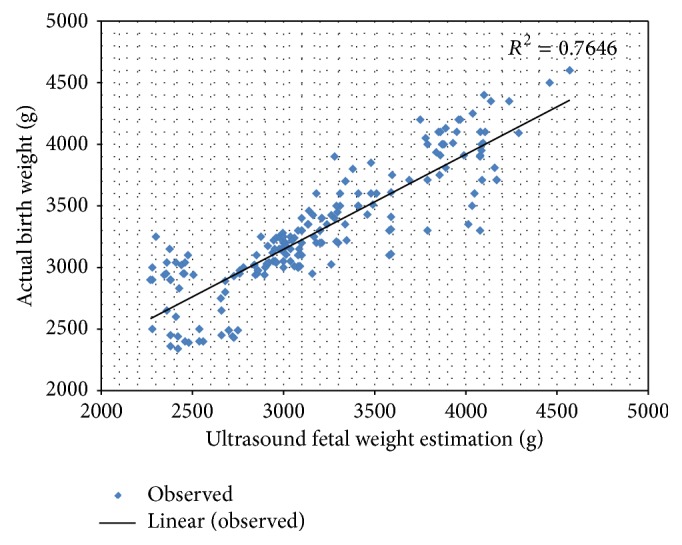
The scatter diagram of ultrasound fetal weight estimation and actual birth weight. Ultrasound method showed positive correlation with actual birth weight.

Figure 2 is the scatter diagram showing the relationship between ultrasound fetal weight estimation and actual birth weight. Ultrasound method of fetal weight estimation showed a positive correlation with the actual birth weight of the fetus after delivery. There is a linear relationship between ultrasound fetal weight estimation and actual birth weight.

The validity of clinical and sonographic fetal weight estimation is shown in Table 3. The sensitivity values for both clinical and ultrasound methods were 75% and 69.4%, respectively, and the difference was not statistically significant (*P* = 0.3447).

**Figure 3:**
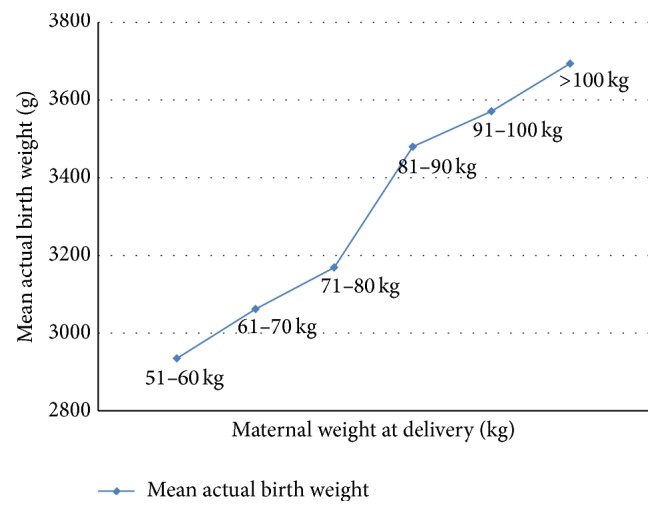
The relationship between maternal weight at delivery and the actual birth weight. The actual birth weight increases as the maternal weight increases.

The relationship between maternal weight at delivery and the mean actual birth weight is shown in Figure 3. There is a positive linear relationship between maternal weight at delivery and mean birth weight of the babies. The mean actual birth weight increased with increase in maternal weight.

The mean errors for both clinical and ultrasound methods were 299 ± 338 g and −101 ± 189 g (Table 4). The mean percentage errors were 9.2% ± 10.44 and −3.108% ± 9.67 for clinical and ultrasound methods, respectively, and were statistically significant (*P* = 0.0000). This means that, in the entire study group, the clinical method significantly overestimated actual birth weight, while the ultrasonic method underestimated it. The mean absolute percentage errors of both clinical and ultrasound methods were 11.16% ± 9.48 and 9.04% ± 7.61, respectively. The mean absolute percentage error was smaller for ultrasonic estimation, although the difference was not statistically significant (*P* = 0.205).

The correlation coefficient for the clinical and ultrasonic methods, compared to actual birth weight, was +0.740 and +0.847, respectively, and results of statistical analysis showed the relationships to be statistically significant (*P* = 0.002).

## 4. Discussion

Accurate prediction of fetal weight has been of great interest in obstetrics. As fetal weight cannot be measured directly, it must be estimated from fetal and maternal anatomical characteristics. Of the various methods, the most commonly used are the clinical and ultrasonographic methods as in this study. Both fetal macrosomia and intrauterine growth restriction increase the risk of perinatal morbidity and mortality and of long-term neurologic and developmental disorders [3, 5]. Identification of intrauterine growth restriction and macrosomia will reduce the chance of fetal morbidity and mortality [3, 5].

The mean actual birth weight in this study was 3,242 ± 508 g. This was similar to the mean actual birth weight of 3,254 ± 622 g reported by Shittu et al. in Ife, Nigeria [11], and slightly higher than 3.08 ± 0.610 Kg in Makurdi, Nigeria [12], and 3.10 ± 1.89 kg in Jos [13]. However, it is lower than 3,568 ± 496 g documented in United Kingdom [14]. The reason may be due to several factors affecting birth weight such as regional and socioeconomic factors [4, 13].

The mean of ultrasonic weight estimation was 3,141 ± 441 g. When the result was compared with actual birth weight, it was found that actual birth weight was not significantly different. Also, the mean of clinically calculated weight of fetus was 3,541 ± 633 g. After comparing it with actual weight of baby after birth, it was found that actual birth weight was also not significantly different from the clinically estimated weight. So it is clear from this finding that the clinical assessment of weight is comparable to ultrasound in prediction of actual birth weight. The finding was in sharp contrast to the study by Ugwu et al. where ultrasound estimation was significantly more accurate than clinical prediction [1]. However, it is similar to the finding obtained in some earlier studies [6, 11, 15].

There was a positive linear relationship between maternal weight at delivery and mean birth weight of the babies in this study. This was also the finding in a study in Ile-Ife [11] and Jos, Nigeria [13]. In this study, clinical estimation had more percentages of sensitivity (75%) and negative predictive value (93.4%) than the ultrasonic estimation, that is, 69.4% and 92.7%, respectively. However, the difference in their sensitivity and negative predictive values was not statistically significant.

The study revealed that clinical estimation of fetal weight is as accurate as the ultrasonographic method of estimation, although, the clinical method overestimated fetal weight and ultrasonic method underestimated it. Three measures of accuracy were used in this statistical analysis and these include the number of estimates within ±10% of actual birth weight, mean percentage error, and mean absolute percentage error. Interestingly, the mean percentage error can be misleading because it is the sum of positive and negative deviations from actual birth weight, thus artificially reducing the difference between actual birth weight and estimated birth weight. It is a measure of systematic error in each method and only takes into account under- or overestimation biases. By contrast, the mean absolute percentage error reflects the variability noted regardless of their direction and, as such, is the best and much more accurate predictor of differences from actual birth weight [11]. Hence, the variation between predicted birth weight and actual birth weight was expressed in the form of mean absolute percentage error in this study.

The study showed that the mean errors for both clinical and ultrasound methods were 299 ± 338 g and −101 ± 189 g. The mean percentage errors were 9.2% ± 10.44 and −3.10% ± 9.67 for clinical and ultrasound methods, respectively. This means that, in the entire study group, the clinical method systematically overestimated actual birth weight, while the ultrasonic method underestimated it. The mean absolute percentage errors of both clinical and ultrasound methods were 11.16% ± 9.48 and 9.04% ± 7.61, respectively. Shittu et al. found that the mean absolute percentage errors by clinical and ultrasound methods were 9.7% and 9.9% and that the error was higher in ultrasonographic method though the difference was not statistically significant [11]. In this study, the mean absolute percentage error was lower in ultrasound method though the difference was not statistically significant. The reason may be due to improvement in skills, knowledge of scanning, and the quality of ultrasound machine in recent time. These results are also consistent with what have been previously observed that the mean absolute percentage error of predicting birth weight varies from 6% to 12% of actual birth weight, and 40–76% of the estimates were within 10% of actual birth weight [16–18]. In the study evaluating the accuracy of fetal weight estimation through clinical palpation using the product of symphysiofundal height and abdominal girth in full-term patients, Raghuvanshi et al. reported a mean absolute percentage error of 12 percent [19] which was very similar to the value obtained in this study.

The correlation coefficients for the clinical and ultrasonic methods in this study, compared to actual birth weight, were +0.740 and +0.847, respectively, and both correlated positively with the actual birth weight. The correlation coefficient for ultrasound estimation is comparable with that of Uotila et al. (0.77) [20] and Shittu et al. (0.74) [11] in their comparison of ultrasonic estimation. The correlation coefficient of clinical estimation is comparable to that of Shittu et al. (0.78) [11] in a similar population.

The accuracy within 10% of actual birth weights was 69.5% and 72% for both clinical and ultrasound estimation of fetal weight, respectively, and the difference was not statistically significant. The finding in this study was comparable to the study by Shittu et al. (2007) which reported that 70% and 69% of estimated fetal weights were within 10% of actual birth for clinical and ultrasound method, respectively, and the difference was not statistically significant [11]. Amritha et al. reported that the rates of estimates within 10% of birth weights were not statistically significant in clinical method and ultrasound methods (67% and 62%, resp.) [21].

The observations imply that there is clearly a role for clinical estimation of birth weight as a diagnostic tool, suggesting that clinical estimation is sufficient to manage labor and delivery in a term pregnancy. Peregrine et al.found no advantage of sonographic estimation over clinical or patients' estimation of fetal weight at term [16]. Furthermore, Nahum and Stanislaw found that the use of ultrasonography was generally no more accurate than prediction that is based solely on quantitative assessment of maternal and pregnancy specific characteristics [22].

The above findings have important implication for developing countries like ours where there is paucity of technologically advanced ultrasound machines capable of doing sophisticated functions such as fetal weight but have experienced clinicians who could perform this function equally well. Routine requesting of costly ultrasound estimates is hardly justifiable when clinical estimation by Dare's method is equally as accurate as ultrasound method and can be quickly carried out at no cost, easy to perform, and a practical tool for predicting birth weight, especially for less experienced examiners [11, 23].

## 5. Conclusion and Recommendations

This study indicates that clinical estimation of birth weight clearly has a role in management of labor and delivery in a term pregnancy. Among term singleton cephalic pregnancies studied, fetal weight estimation using Dare's formula is comparable to ultrasound estimates for predicting the actual birth weight within 10%. This study also revealed that there was no significant difference found between the mean weight obtained through clinical and ultrasound assessments and actual birth weight. Both clinical (Dares formula) and ultrasound methods of fetal weight estimation showed positive correlation with actual birth weight of the fetus after delivery. So it is clear from this finding that the clinical assessment of fetal weight is another good predictor of actual birth weight along with ultrasonic estimation of fetal weight. Clinical estimation of fetal weight is good enough for screening of the birth weight as it has higher sensitivity and negative predictive value than the ultrasonic estimation, while specificity and positive predictive ultrasonic estimation were higher than clinical estimation. It means that clinical estimation in screening for actual birth weight is better. Thus, keeping in view that the clinical procedure by Dare's method is easy to perform, it can be included in routine training of medical personnel.

Recommended based on the findings from this study is that clinical fetal weight estimation should be taught to all health workers. And it is suggested for use as a routine screening tool for all parturients at term and in labor.

## Figures and Tables

**Table 1:** The demographic characteristics of the study population.

Characteristics	Mean (S.D.)	Median	Range	Mean (S.D) LBW	Mean (S.D) NBW	Mean (S.D) MB	*P* value of ANOVA
Maternal age (years)	28.86 (6.355)	27	16–44	24.92 (5.854)	28.78 (6.580)	31.42 (5.073)	0.060
Parity	2.14 (1.737)	2	0–9	1.33 (1.96)	2.04 (1.51)	3.25 (2.32)	0.001^*^
Maternal weight (kg)	72.48 (11.561)	71.00	53–109	63.66 (12.203)	71.93 (10.551)	80.62 (12.011)	0.000^*^
Gestational age at delivery (weeks)	39.5 (1.513)	39	37–42	37.6 (2.068)	39.9 (1.420)	38.9 (1.725)	0.36

*P* value (*P* ^*^ < 0.05) = significant.

LBW: low birth weight.

NBW: normal birth weight.

MB: macrosomic birth.

S.D.: standard deviation.

**Table 2:** The mean actual birth weights and mean fetal weight measured by clinical and ultrasound methods.

	*N*	Mean ± S.D.	Minimum	Maximum	*t*-test	*P* value
Actual fetal weight	200	3,242 ± 508 g	2,350 g	4,600 g		
Clinical fetal weight	200	3,541 ± 633 g	2,381 g	4,924 g	0.453	0.695
Ultrasound fetal weight	200	3,141 ± 441 g	2,270 g	4,590 g	2.259	0.122

*P* value (*P* < 0.05) = significant.

**Table 3:** The validity of clinical and sonographic fetal weight estimation.

Validity	Clinical method	Ultrasound method	*P* value
Sensitivity	75%	69.4%	0.3447
Specificity	78.6%	85.3%	0.269
Positive predictive value.	43.5%	51.0%	0.3215
Negative predictive value.	93.4%	92.7%	0.7742

**Table 4:** The errors and correlation of ultrasound and clinical fetal weight estimation with the actual birth weights of the babies.

Parameter	Clinical ± std.	Ultrasound ± std.	*P* value
Mean error (gram)	299 ± 338 g	−101 ± 189 g	0.0000
Mean absolute error (gram)	362 ± 307 g	293 ± 313 g	0.205
Mean percentage error	9.2% ± 10.44	−3.1% ± 9.67	0.0000
Mean absolute % error	11.16% ± 9.48	9.04% ± 7.61	0.205
Correlation coefficient	0.740	0.847	0.002
Accuracy within 10% of ABW	69.5%	72%	0.755
